# 2025 Content Collections from the IDSA Journals

**DOI:** 10.1093/ofid/ofag032

**Published:** 2026-02-15

**Authors:** Roger Bedimo, Paul E Sax, Cynthia L Sears

**Affiliations:** University of Texas Southwestern Medical Center, Dallas, TX, USA; Division of Infectious Diseases, Brigham and Women's Hospital, Boston, MA, USA; Johns Hopkins University School of Medicine, Baltimore, MD, USA

The past year has been a momentous one in the field of infectious diseases, particularly in the United States. Throughout, the Infectious Diseases Society of America journals have held strongly to the principles of scientific integrity and global health equity while working to encourage and spotlight rigorous and inclusive dialogue. [1] We as editors have also had the pleasure of supporting the development of timely content on topics worthy of attention from infectious diseases clinicians and scientists. As the new year begins, we wish to take a moment to draw your attention to some of that content.

## Special Collections

The IDSA journals hosted and collaborated on several collections in 2025, including:

**Healthcare sustainability through the infectious diseases lens.** A collaborative effort of the *Journal of the Pediatric Infectious Diseases Society* and *Open Forum Infectious Diseases*, this collection explored the interconnections among infectious diseases, climate change, and sustainability. Topics included strategies for reducing antimicrobial waste; sustainable infection prevention and control practices; outpatient infectious diseases healthcare sustainability; and international effects of waste management practices. [2, 3]

**Infectious diseases in people who use drugs.** This *Open Forum Infectious Diseases* collection investigated approaches to the syndemic of infectious diseases and substance use disorders. Papers discussed how to prevent, treat, and manage these infections by using patient-centered care and addressing structural factors. The collection also featured updates on emerging infections and the care of xylazine-associated wounds. [4]

**Neurovirulent infections.** A collaboration between *The Journal of Infectious Diseases* and *The Journal of the Pediatric Infectious Diseases Society,* this series explored the nuances of pathogenic mechanisms underlying neurologic infections, with a goal of encouraging the development of optimal pathogen and host-directed therapeutic strategies. Experts reviewed the most up-to-date information on the neurovirulence of Group B *Streptococcus*, *Streptococcus pneumoniae*, cytomegalovirus, Zika virus, enterovirus, herpes simplex virus, arboviruses, malaria, toxoplasmosis, cryptococcus, and tuberculosis. [5, 6]

**Planetary health.** This *Open Forum Infectious Diseases* collection was dedicated to the interplay between natural and built environments and infectious diseases. Topics included prevention of infectious disease through environmental intervention; agriculture and antimicrobial resistance; ecological factors involvement in infectious diseases; and emerging and expanding zoonotic and vector-borne diseases. [7]

**Tuberculosis isolation.** This joint collection from *Clinical Infectious Diseases* and *The Journal of Infectious Diseases* was developed in response to new guidelines from the National Tuberculosis Coalition of America on the isolation of TB patients in community settings. The collection brings together clinical guidelines for the use of isolation in TB management, reviews of the history of isolation as a public health intervention and of the determinants of TB infectiousness, and a commentary on an ethics-informed public health guideline framework. [8, 9]

**Vaccine sciences.** At a critical time in the development of vaccine science and policy, this collection from *The Journal of Infectious Diseases* brings together perspectives from leading experts on a wide range of topics, including global health and access to vaccines, systems vaccinology, and vaccine hesitancy. The collection looks back at advancements over the past decade and ahead to future opportunities in the field. [10]

Looking ahead to 2026, you can look forward to forthcoming collections on antimicrobial resistance, new point-of-care diagnostics, cryptosporidiosis and artificial intelligence in infectious diseases, with more topics in development. We will share our annual “Best of the IDSA Journals” collection in January 2026, capturing a selection of the most timely and interesting research published in our journals in the past year. In addition, we will continue to build on regular features in our journals, including CID’s State of the Art Reviews, Clinical Dilemmas in ID, and Ethics Rounds and JID’s ID Translational Science Updates and Viewpoints.

## Our Thanks

As editors-in-chief, we also must take this opportunity to thank the large community of infectious diseases researchers whose knowledge and time make the publication of our journals possible. We are grateful for the contributions of our fellow editors, our dedicated peer reviewers, and the authors who share their crucial findings and insights with our readers. We look forward to working with all of you to advance the science and practice of infectious diseases in the year to come.
